# Passive wheels on legged robots: a survey

**DOI:** 10.3389/frobt.2026.1857985

**Published:** 2026-06-17

**Authors:** James Florin Petri, Gerard Lacey

**Affiliations:** Department of Electronic Engineering, Maynooth University, Maynooth, Ireland

**Keywords:** low friction, passive wheels, robot, skating, skiing, unactuated, underactuated, underdetermined

## Abstract

In recent years, highly dynamic robots have seen a significant rise in both consumer and research applications. Much of the focus on legged robots stems from their ability to adapt to a world built for humans, but energy inefficiency and battery capacity remain significant challenges to widespread adoption. Wheel-legged hybrid locomotion is an increasingly popular solution, as robots combine the energy efficiency and speed of wheels with the versatility and adaptability of legs. Most of the literature focuses on the actuated or active-wheel variant because it is simpler to control. However, recent advances in control strategies and computing power have made the advantages of unactuated or passive wheel-legged robots, such as their mechanical simplicity, low weight, and high energy efficiency, accessible. In this survey, we provide a comprehensive analysis of the unactuated wheel-legged hybrid robot literature, together with similar systems, to review the alternative implementations and design techniques in the context of the inherent challenges of controlling a legged robot that has passive skates on its feet. We aim to outline the critical factors that determine the system’s viability and propose some directions for future research.

## 1 Introduction

Legged robots have become increasingly common in both research and commercial settings, facilitated by recent developments in modelled and learned control, mechanical designs, and computation power. However, challenges in energy efficiency, real-world robustness, adaptability, autonomy, and cost continue to limit their widespread adoption beyond niche applications (Staniaszek et al., 2024; Hutter et al., 2016; Kulkarni et al., 2022; Fiorucci et al., 2023; Bi et al., 2025).

Interest in hybrid wheel-legged locomotion has also grown significantly as it brings together the legged locomotion domain, defined by adaptability and manoeuvrability on complex and uneven terrains, and the wheeled locomotion domain, characterised by energy efficiency, stability, and high velocities on mostly flat and predictable terrains. Hybrid wheeled-legged robots result in a versatile platform capable of traversing a wide range of environments (Bjelonic et al., 2018; Chen et al., 2021; Yang et al., 2023a; Geilinger et al., 2020; Pan et al., 2024; Chen W.-T. et al., 2025; Shen et al., 2025). Most of these wheeled-legged robots have actuated or driven wheels (Lu et al., 2013; Geilinger et al., 2020; Pan et al., 2024; Bjelonic et al., 2021; Belli et al., 2021; Arnold et al., 2025; Chen C. et al., 2025). However, interest in passive wheeled variants (also referred to as unactuated or skating) has increased recently due to various advancements in novel control strategies and increased capacity of onboard computing power (Bjelonic et al., 2018; Yang et al., 2023a; Cho and Kim, 2024; Chen et al., 2023; Hung et al., 2025).

The domain of active wheel-legged robots has been extensively explored, yielding impressive results. Multiple configurations have been explored, with the most popular ones being bipedal and quadrupedal. Examples include ETH’s ANYmal on Wheels^1^ or the Boston Dynamics’ Handle^2^, which typically use high-torque actuators to drive the wheels directly. This approach allows multiple gaits, including high-speed rolling, stepping, jumping, and overcoming larger obstacles. These dynamic capabilities are enabled by the direct controllability of the actuated wheels. However, mechanical complexity, high power consumption, and the significant additional weight due to the extra actuators and drivetrain components are challenges to system cost and reliability (Lu et al., 2013; Geilinger et al., 2020; Pan et al., 2024; Bjelonic et al., 2021; Belli et al., 2021; Arnold et al., 2025; Chen C. et al., 2025; De Luca et al., 2021).

In contrast, passive wheel-legged robots use unactuated wheels, achieving locomotion by moving the limbs to induce a rolling motion in the wheels, similar to a human on a skateboard or rollerblades. This design reduces mechanical complexity, lowers leg mass, and improves energy efficiency during sustained travel on smooth surfaces. Early implementations such as the “Roller-Walker” (Endo and Hirose, 1999) and more recent platforms like the Q-Skater (Chen et al., 2024), demonstrate the feasibility of this concept, with examples in the literature achieving a 70%–80% lower Cost of Transport (CoT) while maintaining stable gaits. However, the core challenge lies in the non-holonomic constraints imposed by the wheels and the resulting underactuated dynamics, making stable and agile locomotion a complex control problem (Bjelonic et al., 2018; Cho and Kim, 2024; Endo and Hirose, 1999; 2012; Yang et al., 2023a; Chen et al., 2024; 2023).

Multiple skating platforms have been developed, varying in the number of legs and their dexterity. Bipedal skating robots are a significantly underactuated and a highly unstable system for which generating balanced, multi-directional motion is exceptionally difficult. As with humans, they use both torso and arms while roller skating, and as a result, humanoid robots need to successfully integrate dynamic whole-body control (WBC) into the skating gait, which remains an active research domain. Even so, there are numerous examples of bipedal robots that skate in the literature (Xu et al., 2008; Itabashi and Kumagai, 2010; Jo et al., 2008; Gu et al., 2026). With the addition of more legs, improved dynamically or statically stable gaits can be achieved, simplifying the implementation. The most common skating platform is the quadruped as it offers a good ratio between stability and efficiency (Endo and Hirose, 1999; Bjelonic et al., 2018; Chen et al., 2024; Geilinger et al., 2020). Other configurations, such as skating tripods and hexapods, are not as common (Hung et al., 2025; Yang et al., 2023a).

This review aims to offer the reader an in-depth analysis of passive wheeled-legged locomotion, control, and performance, with a focus on skating robots. Section 2 presents the most influential designs, implementations, and approaches with the aim of analysing the relevant literature and classifying it based on the leg configuration. The section also provides an overview of propulsion methods. At the end of the section, Table 1 is presented, containing all the discussed robots together with relevant deployment metrics and system capabilities. Section 3 analyses the performance of learned controllers relative to model-based approaches on skating robots, aiming to outline possible future directions for the domain. Finally, the discussion and conclusion sections provide an analysis of the literature, a comparison to other related fields, and future development options.

**TABLE 1:** A table of legged skating robots, split vertically based on the number of legs (2, 4, and other).

References	T	MV	H	W	D	WD	SC	PM	LC	MC	ST	RT
Xu et al. (2008)	1	0.2	0.45	3.8	6	20	Q	1		✓		
Jo et al. (2008)	1	/	0.23	1.7	6	/	Q	2		✓		
Kumagai and Tamada (2008)	1	0.24	0.4	/	6	/	Q	1,7				
Itabashi and Kumagai (2010)	1	0.33	1.45	60	6	/	Q	1		✓		
Ishihara et al. (2020)	1	/	/	/	/	/	Q	1,8		✓	✓ *	
Hashimoto et al. (2008)	1	0.4	1.43	71	6	/	I	1,7		✓		
Iverach-Brereton et al. (2014)	1	0.03	0.46	2.9	6	/	Q,I	2		✓		
Gu et al. (2026)	1	1.0	1.4	38	6	62	I	1	✓			
Yoneyama et al. (2009)	4	3.0*	0.6	3.6	6	NA	NA	8		✓	✓ *	
Han et al. (2020)	4	5.0*	1.32	26	6	NA	NA	8		✓	✓ *	
Park et al. (2022)	4	/	1.23	30	6	NA	NA	8		✓	✓ *	
Takasugi et al. (2016)	6	0.5	1.54	58	6	NA	NA	2		✓		
Takasugi et al. (2019)	1	1.03	1.88	127	6	/	Q	1		✓		
Takasugi et al. (2019)	6	1.0	1.88	127	6	NA	NA	2		✓		
Han et al. (2026)	6	/	1.3	35	6	NA	NA	2	✓			
Endo and Hirose (2012)	2	2.27	0.25	37.6	4	/	S	1,7		✓	✓	
Kalouche et al. (2015)	2	0.5	/	/	4	/	S	1,7		✓		
Geilinger et al. (2020)	2	0.97	/	/	4	/	I	1		✓		
Bjelonic et al. (2018)	2	0.34	/	/	3	/	S	4		✓	✓ *	
Chen et al. (2021), Chen et al. (2022)	2	0.49	0.45	30	3	76	S	3		✓		
Chen et al. (2023)	2	0.6	0.3	18	3	76	S	3,4,7		✓		
Chen et al. (2024)	2	0.4	0.3	18	4	74	S	1,7		✓		
Bellegarda et al. (2018)	2	0.5	/	/	6	/	S	1		✓		
Cho and Kim (2024)	2	0.83	/	/	3	/	S	3	✓			
Wang et al. (2026)	2	2.0	/	/	3	/	S	3	✓			
Xu et al. (2024)	7	0.7	0.48	8.9	3	NA	NA	2		✓		
Liu et al. (2025)	7	/	0.4	12	3	NA	NA	2	✓		✓	✓
Yoon et al. (2026)	7	/	0.4	12	3	NA	NA	2	✓			
Yamaguchi et al. (2025)	2	/	/	19.6	3	64	S	1		✓		
Li et al. (2017)	2	0.38	0.23	/	2	/	S	2,9		✓	✓ *	
Wei et al. (2025)	2	4.2*	/	1.5	3	34	S	8		✓	✓ *	
Yang et al. (2023a), Yang et al. (2025)	2	8.2	22	/	3	/	S	5,6		✓	✓	✓
Yang et al. (2023b), Yang et al. (2024)	5	7.0	24	0.7	3	NA	NA	5,6		✓	✓	
Li et al. (2025)	5	/	24	0.7	3	NA	NA	5,6	✓		✓	
Zhai et al. (2016)	3	0.4	/	/	3	/	S	5		✓		
Chitta et al. (2004)	3	/	/	6	3	19	C	5		✓		
Hooks et al. (2020)	3	2.1	/	17.9	3	/	C	5		✓		
Hung et al. (2025)	3	0.56	0.35	4.5	3	25	C	2,7		✓	✓ *	✓

The listed metrics represent only real-world performance, with simulation results omitted, and “/” denotes unavailable data. The columns use the following notation: Reference indicates the cited work; T denotes the system type (1-skating biped, 2-skating quadruped, 3-other skating configuration, 4-skiing biped, 5-other skiing configuration, 6-skateboarding biped, 7-skateboarding quadruped); MV, is the maximum reported velocity in m/s (rounded to two decimals), where “*” indicates downhill velocity; H is the robot height in meters (rounded to two decimals); W is the robot weight in kilograms (rounded to one decimal); D is the degrees of freedom per leg; WD, is the wheel diameter in millimeters (marked NA, if not applicable, e.g., skiing systems); SC, is the skate type (Q-quad skates, I-inline skates, S-single wheel, C-caster wheels); PM, is the propulsion method (1-swizzle/dexterous steering, 2-push-glide, 3-mobility locking, 4-claw/cover, 5-push legs, 6-fan, 7-transformable legs, 8-gravity, 9-braking); LC, indicates the use of a learned controller; MC, indicates the use of a model-based controller; ST, indicates validation on sloped terrain (”*” denotes no uphill demonstrations); and RT, indicates validation on rough terrain (e.g., uneven ground, stairs, or obstacles).

This survey follows the PRISMA guidelines for systematic reviews^3^. The literature search was conducted up to 19 March 2026, across multiple platforms (e.g., IEEE Xplore, Google Scholar, arXiv, GitHub, Google Search, YouTube). The search used combinations of keywords such as, but not limited to, “legged robots”, “passive wheels”, “skating X” (X replaced with leg configuration, e.g., quadruped), “roller feet”, “skating locomotion”, “hybrid leg-wheel robots”, “skiing robot”, “rollerskating/rollerblading robot”, while utilizing search operators and search filters to narrow/widen the search space. After removing duplicates, 198 studies were screened based on titles, abstracts, and figures, followed by full-text review, resulting in 84 accepted works. Each accepted paper was run through Inciteful^4^ for reviewing citation graphs and to identify possibly missed publications, which resulted in the final 92 references (using the same evaluation order). Papers were accepted if they demonstrated skating on a real robot and excluded if the results were only presented in simulation, with the exception of highly novel simulated systems. If multiple similar implementations are identified, priority is given to the oldest, followed by the most cited papers, with the remainder excluded. If skating was implemented on a standard legged robotic platform, a reference or footnote of the platform is added. Significant papers on modelled and learned controller approaches, standard legged locomotion, and active wheel-legged hybrid locomotion were included and cited if relevant to skating or to future developments or trends in the space. Selection is based on pioneering papers, high citation count and overall community adoption and impact in the space. Finally, in some cases, footnotes are included to complement the arguments or analysis provided for various accepted references, or to demonstrate skating.

## 2 A background on skating robots

This section provides a comprehensive review of skating/sliding locomotion for legged robots, organized by the leg configuration, and covering the performance of different robotic platforms in the locomotion domains of wheel-based skating, ice skating, skiing, and to a certain extent, skateboarding. There are subsections dedicated to bipedal and quadrupedal configurations, followed by one dedicated to other configurations. The last subsection offers an overview analysis of all the described propulsion methods. All the systems are organized in Table 1, which summarizes the main performance metrics and system observations.

### 2.1 Bipedal skating robots

**FIGURE 1:**
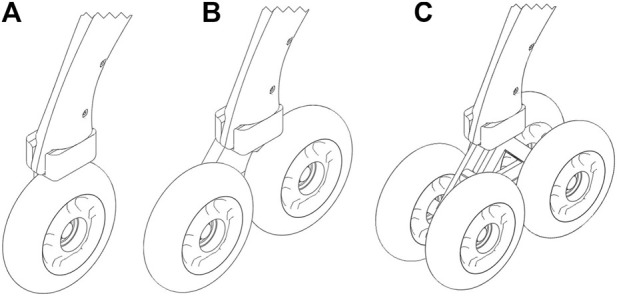
Skate configurations: **(A)** single wheel per leg, offering high manoeuvrability at the cost of reduced stability; **(B)** inline skates, which provide improved stability, particularly at higher speeds, but are more difficult to steer due to the sliding constraint; and **(C)** quad skates, which offer exceptional stability but are heavier, harder to steer, and less effective on uneven terrain without an “ankle” roll joint.

Bipedal/humanoid robots are inherently unstable while moving, requiring advanced modelling and physics considerations to ensure the robot does not fall over. Adding active wheels to the feet can increase stability as the wheels can rapidly correct any sensed error. Skating bipedal robots are not as common as their active counterpart; however, there are still plenty of examples in the literature (Xu et al., 2008; Itabashi and Kumagai, 2010; Ishihara et al., 2020; Gu et al., 2026). Most active wheeled bipeds utilize a single wheel per leg (Figure 1A), however, this configuration is less common for skating bipeds, as inline and quad skates offer a more favourable trade-off between stability and performance.

Roller-skates (quads) have 4 wheels placed in a rectangle (Figure 1C), allowing for more stability on each leg. Multiple humanoid/bipedal robots demonstrated roller-skating gaits and implementations (Xu et al., 2008; Jo et al., 2008; Kumagai and Tamada, 2008; Itabashi and Kumagai, 2010). Xu et al. (2008) successfully implemented skating on BISR, their 0.45 m tall bipedal robot, achieving speeds of around 0.2 m/s by having the swizzle gait analytically derived with generalized Lagrange-Maggi equations and replayed on the hardware with no online feedback. Jo et al. (2008) generated joint trajectories using a Zero Moment Point (ZMP) based planner for their 1.714 kg 0.228 m tall humanoid. The robot uses a stroke-like skating gait, alternating between a pushing leg (for propulsion) and a gliding leg (for stability and forward motion). Unfortunately, no speed metrics were provided for its implementation.

**FIGURE 2:**
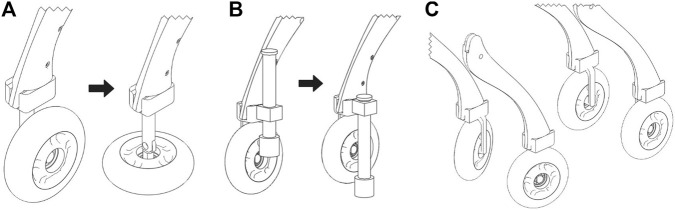
**(A)** illustrates a simplified model of a transformable leg, in which the wheel can be rotated beneath the leg to function as a foot (Endo and Hirose, 1999; Kalouche et al., 2015). **(B)** Illustrates another transformable leg option, where a contact point is lowered under the level of the wheel. An alternative would be for the wheel to raise. **(C)** Demonstrates mobility locking as a propulsion strategy, where the hind legs are angled outward. When all legs remain stationary, the robot is unable to roll, however, lifting or rolling one of the hind legs unlocks mobility. When combined with pushing actions, this mechanism enables stable propulsion, as demonstrated by Cho and Kim (2024).

Kumagai and Tamada (2008) also implemented a Roller-skate swizzle gait on their 0.4 m tall biped by precomputing the joint positions, achieving speeds of up to 0.24 m/s. Notably, their platform employs transformable feet that can swap between standard soles and wheels (simplified visualization in Figures 2A,B). Additionally, their skate wheel axle mechanism allows the wheels to follow the curvature of the ground in non-flat environments. Itabashi and Kumagai (2010) continued their previous work (Kumagai and Tamada, 2008) by developing Zephyr and adapting it for skating. The robot was 1.45 m tall and weighed around 60 kg. They implemented the swizzle gait using 5^th^ order Bezier curves to model the feet trajectories, achieving a speed of around 0.33 m/s. Sony presented QRIO at IROS 2004, which was shown skating on flat and sloped terrain in a stroke-like gait. However, there is very little information on it online^5^. Ishihara et al. (2020) also implemented roller-skating on a biped using a hierarchical model predictive controller with a reflex-based stabilizer, demonstrating standing on flat ground and downhill skating on the real robot, while more agile manoeuvrers were only shown in simulation.

Roller-blades, also referred to as “inline skates” (Figure 1B), allow for greater speeds, more adaptability, and better roll control on non-flat terrains. However, stability can be a concern when only one leg is in contact with the ground. Hashimoto et al. (2008) adapted their bipedal robot, WL-16R, for skating (For the combined system WL-16R/WS-3) (Sugahara et al., 2004; Hashimoto et al., 2005). The robot used Stewart Platforms for legs, weighing 71 kg and being 1.431 m tall. The inline skates had rubber pads that were used as brakes when rolling the skate to the side. Swizzling locomotion was realized by dynamically modifying the periodic foot trajectories in real-time based on measured foot reaction forces, achieving speeds of up to 0.4 m/s. Iverach-Brereton et al. (2014) modified the DARwIn-OP humanoid platform (Ha et al., 2011) for ice and roller skating. The robot approximately weighed 2.9 kg and was about 0.46 m tall. An alternating push-off glide skating gait was implemented by executing precomputed joint position trajectories, achieving around 0.03 m/s while ice skating and around 0.026 m/s while skating, which are slower than the other approaches and platforms mentioned above.

Gu et al. (2026) conducted a comprehensive experimental comparison of skating (inline skates) to walking locomotion on a bipedal humanoid. By using a Deep Reinforcement Learning (DRL) based controller, their robot, “SKATER”, achieved velocities of 1 m/s, reduced Cost of Transport by 64.34% relative to walking, and lowered contact force impact intensity by 75.86% (the latter validated in simulation only), while also maintaining a 100% traversal success rate across multiple flat terrain types with varying friction. While in simulation, the robot was capable of reaching 2 m/s, the hardware experiments were limited to 1 m/s due to safety concerns regarding the reliability of braking at higher speeds. The control policy was trained in IsaacGym (Makoviychuk et al., 2021) using Proximal Policy Optimization (PPO) (Schulman et al., 2017) in large-scale parallel simulation environments relying on an implicit gait formulation, where skating behaviours are promoted through reward terms encoding physical consistency, inter-leg symmetry, and non-holonomic wheel constraints rather than explicit phase timing or reference trajectories. The learned policy presented robust sim-to-real transfer and, to our knowledge, considerably outperforms traditional model-based and trajectory-tracking controllers reported in the literature for bipedal skating. However, the study does not report performance on rough or discontinuous terrain, does not evaluate performance on slopes, and focuses primarily on continuous swizzle gaits on relatively planar environments, leaving these aspects as open limitations.

Skiing bipedal robots are similar in many ways to the skating ones; they differ in their increased stability due to the reduced likelihood of falling forward or backwards and in their ability to achieve relatively high speeds when moving downhill due to significantly lower ground friction (Yoneyama et al., 2009; Han et al., 2020; Wu et al., 2021; Park et al., 2022; Boboc, 2025). Yoneyama et al. (2009) developed a 0.6 m tall bipedal skiing robot that managed to achieve speeds of up to 3 m/s going downhill through open-loop control, where each joint angle was programmed as a sinusoidal function with pre-tuned amplitude and phase shift. Another example is RoK-2, developed by Han et al. (2020), which uses a model-based controller with predefined skiing motion primitives and ZMP-based balance control. They achieve stable downhill skiing at stated speeds of 3–5 m/s, which was demonstrated at the 2018 Ski Robot Challenge, where it won bronze^6^ ^,^ ^7^.

Takasugi et al. (2016) designed a skateboarding controller for the HRP-2 (Okada et al., 2005), a 1.54 m tall humanoid robot that weighs 58 kg. They used a real-time ZMP based controller with slip aware foot force planning and QP-based stabilization to achieve 0.5 m/s. In later research, Takasugi et al. (2019) implemented walking, roller skating (quads) and skateboarding on their humanoid platform, JAXON (Kojima et al., 2015), using a 3D centre of mass (3D COM) trajectory optimization with contact wrench cone (CWC) and divergent component of motion constraints to achieve stable locomotion, reaching walking speeds of 0.6 m/s, skateboarding speeds of 1.0 m/s and roller skating speeds of 1.03 m/s. Notably, the skateboarding and skating velocities are higher than the walking speed. However, the command error rate was reported to be considerably higher for roller skating.

More recently, Han et al. (2026) presented HUSKY, a learning-based framework that enables a humanoid robot to perform complete skateboarding manoeuvrers, including mounting, pushing, steering, and dismounting, validated on a Unitree G1^8^. Their approach models the coupled humanoid-skateboard dynamics by explicitly incorporating the kinematic relationship between board tilt and truck steering, and by leveraging motion priors to learn natural pushing behaviours, alongside a physics-guided strategy for lean-to-steer control, thereby providing a more comprehensive and robust controller. While they did not provide deployment velocity metrics, they achieved 1.0 m/s in simulation. Finally, there are some implementations where passive wheels are used to eliminate the swing phase in the walking gait of a biped (Kimura et al., 2020). However, as these are not examples of true skating, they are not expanded upon here.

### 2.2 Quadrupedal skating robots

The research conducted by Endo and Hirose (1999), Endo and Hirose (2000), Endo and Hirose (2008), Endo and Hirose (2011), Endo and Hirose (2012) on^9^ ^,^ ^10^ the Roller-Walker demonstrated the efficiency and performance of a skating quadruped while also exploring transformable legs for more terrain adaptability (Figure 2A). Early work established baseline mobility, achieving manoeuvrability on a flat surface using simple open-loop joint-position control with offline-optimised fixed trajectories (Endo and Hirose, 1999; 2000). The controller was later improved by introducing an asymptotic parameter adaptation method that used velocity error feedback to adjust the leg trajectory online, enabling robust locomotion on surfaces with varying friction and slope climbing on a 3° incline (Endo and Hirose, 2008). In their final experimental studies, the authors validated the platform’s peak performance using fully optimised trajectories, achieving a maximum speed of 2.27 m/s and an eightfold improvement in energy efficiency compared to a conventional crawl gait (Endo and Hirose, 2011; 2012). Their research and results were instrumental in validating the performance increase prospects of passive wheels on legged robots and are widely considered one of the pioneering works in the space. Kalouche et al. (2015) demonstrated a modular extension of the Roller-Walker concept, using passive wheel-leg locomotion within a highly reconfigurable series-elastic actuator (SEA) design^11^. Their “Snake Monster” platform could be rapidly reconfigured across multiple morphologies, with the quadruped roller-walker using ankle-mounted passive wheels to switch between walking and efficient rolling. This work focuses on modularity as a key enabler for hybrid locomotion, allowing a single hardware system to balance robustness, efficiency, and task adaptability.

**FIGURE 3:**
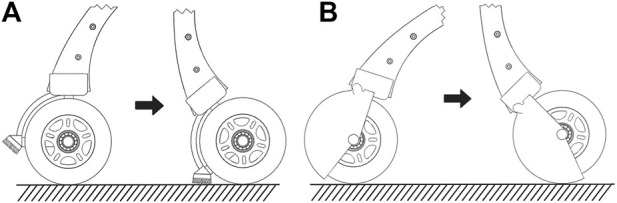
The claw-based propulsion method used in underactuated quadrupedal skating, in which the “claw” makes contact with the ground at a specific tibia orientation. **(A)** Illustrates the standard rubber-pad claw employed by Bjelonic et al. (2018), while **(B)** shows a compressible rubber wheel cover that can be used either for propulsion or, with reversed knee bending, as a conventional foot. This latter design is analogous to a transformable wheel-leg system, as demonstrated by Chen et al. (2023).

Geilinger et al. (2018), Geilinger et al. (2020) developed a computational tool that can be used for generating stable and kinematically valid motion for a variety of legged robots designed using their framework (including standard legs and active and passive wheels) (Geilinger et al., 2018). They validated their approach on several prototypes, including AgileBot, running on actuated wheels, and SkaterBot, equipped with two passive inline wheels per leg. By pitching its ankle joints, SkaterBot could also operate as SwizzleBot, using only one wheel per leg for a swizzling motion. All the robots had the same legs with 4 degrees of freedom. Their experiments showed that the SkaterBot (0.9677 m/s) and SwizzleBot (0.6897 m/s) were faster than their active counterpart, the AgileBot (0.4762 m/s) (Geilinger et al., 2020). This performance advantage is attributed to the efficient, dynamics-based propulsion enabled by passive wheels. Bjelonic et al. (2018) added passive wheels and ice skates to the ANYmal quadruped with “claws” used for generating propulsion, visualized in Figure 3A. They achieved stable locomotion and a reported CoT drop of 80% w.r.t. the standard platform, however the achieved speeds that were quite low (0.342 m/s) and the implementation does not provide much demonstrated terrain adaptability other than going down a 10° slope^12^. Both Geilinger et al. (2020) and Bjelonic et al. (2018) also implemented ice skating on the robots, performing well and demonstrating the transferability of skating robots to real-world terrain challenges^13^.

Chen et al. (2021), Chen et al. (2022) built and tested skating on a 30 kg quadrupedal skating robot with 3 DoF per leg. As a result of the underactuation, the robot could not steer its wheels to generate propulsion. Therefore, they rotated the approach angle (initial yaw angle relative to the robot’s x-axis) of the wheels 20° outwards. By lifting and synchronising the legs, pushing motions can be formed^14^ (Figure 2B). By implementing offline-designed roller-skating foot and body trajectories executed with online ground-reaction-force distribution and joint torque control, they successfully achieved skating with a cost of transport as low as 0.09, which is 85% lower compared to the standard trot gait (Chen et al., 2019; 2021; 2022). Chen et al. (2023), Chen et al. (2024) continued their work by developing QSkater and QSkater-E^15^, which are smaller and more polished versions of their previous robot. Both versions use one wheel skates that have a cover on one side that can be used either for walking normally or as a brake/for propulsion (see Figure 3B). QSkater-E adds a 4^th^ DoF to each leg controlling the wheel’s angle and allowing for a direct comparison between the two versions. Interestingly, while both versions have a considerable decrease in CoT relative to standard walking, the QSkater-E’s CoT is 25% higher relative to its 3 DoF version. They concluded that this is a result of the additional energy requirements needed for maintaining constant wheel yaw angle while forces are pushing against the joint. Nevertheless, the added DoF lowered the turning circle and increased the manoeuvrability of the robot (Chen et al., 2023; 2024).

Bellegarda et al. (2018) validated passive wheel skating on the RoboSimian quadruped, featuring 6 actuated degrees of freedom per limb, using an open-loop controller with offline planning. Stable skating was achieved on 3 and 4 wheels through phase-offset leg periodic motions for propulsion. Bellegarda and Byl (2019) showed the use of RL to improve performance using a simplified Cartesian-space model and proximal policy optimization with kinematic priors. This significantly reduced training time and sample complexity while producing a more robust and adaptive skating policy in simulation than the joint-space RL or hand-designed trajectories. Their work demonstrated that incorporating domain knowledge (e.g., forward/inverse kinematics) into the RL framework allowed the agent to learn stable skating gaits using reduced compute resources, substantially outperforming open-loop trajectories in success rate over varying terrain and friction. However, no hardware deployment metrics were provided.

Cho and Kim (2024) designed a model-based RL skating policy for a modified Unitree Go1^16^. The policy was trained in IsaacGym, with ground reaction forces computed using a simplified Single Rigid Body Dynamics model. The quadruped had three actuated DoF per leg, with the wheels on the hind legs angled 20° outwards, enabling propulsion by strategically lifting the angled legs to lock and unlock the robot’s mobility (Figure 2B). They achieved speeds of 0.83 m/s and a 73.9% decrease in the CoT relative to the standard Go1 trotting gait^17^. Notably, their implementation presented exceptional manoeuvrability, stability, and adaptability relative to other modelled approaches, highlighting the advantages of RL in underactuated locomotion systems. However, the performance on rough/sloped terrain was not presented. More recently, Wang et al. (2026) proposed a framework for quadrupedal skating with passive wheels, where each foot is equipped with a configurable 3D printed roller skate, enabling the exploration of the optimal mobility locking design alongside training a locomotion policy. Using a bi-level optimization loop in IsaacLab, Bayesian Optimization was used in the outer loop to search wheel yaw angles and Reinforcement Learning (PPO) was used in the inner loop to train motor policies. They achieved efficient and versatile skating on a Unitree Go1, with their best design reducing the cost of transport (CoT) by 14.6% relative to their baseline. Based on their system and exploration, the optimal mobility-locking configuration has the front legs at ± 37° inwards, while the hind legs are set at ± 10° inwards. Additionally, they presented emergent behaviours such as a hockey stop (rapid braking by turning sideways), which cut stopping time by approximately 50% from an initial speed of 2 m/s, and a self-aligning motion that automatically reorients the robot for optimal energy efficiency. While the overall performance is impressive, the approach lacks validation on off-road terrain, provides limited detailed velocity data, and incurs a high computational cost. Boston Dynamics’ Spot is also briefly shown performing a “hand-stand” and a front flip while balancing on roller skates, using an RL behaviour policy. However, there is no published information on the implementation specifics^18^.

Skateboarding quadrupeds have also been reported in the literature, with interest driven by their flexibility in mounting and dismounting skateboards for a more energy-efficient mode of transport (Xu et al., 2024; Liu et al., 2025). Xu et al. (2024) used an optimisation-based control pipeline with offline trajectory generation and online linear MPC, which achieved precise steering control and speeds of up to 0.7 m/s on flat ground. Liu et al. (2025) propose a reinforcement learning framework (DHAL) that integrates discrete-time hybrid automata with learned dynamics and a multi-critic policy for mode-switching locomotion. This demonstrated effective skating with a Unitree Go1 on both flat and rough terrain, including grass, snow, stairs, slopes, and ramps. While Xu et al. (2024)’s optimization approach provides interpretable dynamics for precise steering control, Liu et al. (2025)’s learning method demonstrates superior terrain robustness through autonomous mode discovery without requiring predefined gaits. Yoon et al. (2026) propose a phased conditioned reinforcement learning controller for skateboarding, which uses proprioceptive sensing and the ground-facing camera of the Go1 for board stabilisation, and was successfully deployed to the real robot. The simulation results demonstrated stable and coordinated phase-dependent locomotion behaviours, including consistent board tracking, recovery from perturbations, and smooth transitions across motion phases, while also achieving 1.5 m/s. However, only limited quantitative deployment metrics were provided beyond the successful transfer. Ding and Chen (2012) presents an alternative mechanical approach in which the robot skates by periodically varying its body width (similar to an accordion), causing its passively wheeled feet to angle outward and inward, thereby generating forward propulsion. Other noteworthy skating quadrupeds and control approaches have also been reported in the literature, but not expanded on in this review due to their similarity to the other mentioned works (Yamaguchi et al., 2025; Li et al., 2017; Wei et al., 2025).

### 2.3 Other skating configurations

**FIGURE 4:**
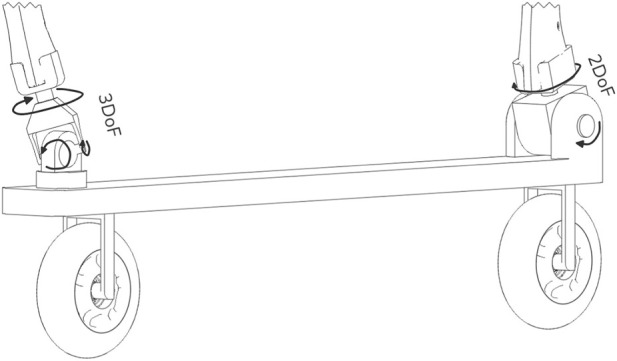
A simplified representation of the “skateboards” used by Yang et al. (2023a), Yang et al. (2025), where the front leg is connected to the board with a 3DoF joint, while the hind leg is connected with a 2DoF joint. The skis used by Yang et al. (2023b), Yang et al. (2024) have the same connection method.

Yang et al. (2023a), Yang et al. (2025) adapted a 6 legged robot for skating by connecting the front and back legs with 2 wheeled “skateboards” (with 5 DoF per skateboard, visualized in Figure 4), leaving the middle legs for velocity and direction control. Using their custom gait, implemented via a Skating Motion Planner (SMP) and a Centroid Balance Controller (CBC) that uses quadratic programming for force optimization, they achieved speeds of up to 2.17 m/s and a 95% command tracking accuracy while also presenting exceptional direction control, stability, and adaptability to uneven terrain (Yang et al., 2023a). In later research, Yang et al. (2025) modified the robot by adding passive wheels to the middle legs as well and a ducted fan to the back of the robot, with the aim of experimenting with fan propulsion for skating. They implemented a PID-based speed controller for the ducted fan, coupled with an adaptive body-position adjuster based on thrust sensing, and separate planners for slow and rapid braking behaviour. They reported acceleration up to 8.2 m/s, braking to a full stop in less than 1 s, and maintaining constant speed while moving. They also mention going uphill, but do not provide a slope angle, nor do they provide any metrics for the energy consumption for the fan.

Yang et al. (2023b), Yang et al. (2024); Wang et al. (2025); Li et al. (2025) modify the hexapod described above for skiing, with the front and back legs connected to skis (5 DoF per ski), while the middle legs have ski sticks at the end. Yang et al. (2023b), Yang et al. (2024), Yang et al. (2025) use the same ducted fan previously mentioned for additional skiing propulsion. Yang et al. (2023b) use a hierarchical strategy similar to its skating variant, combining a trajectory planner with a centroidal balance controller and similar quadratic programming to compute optimal ground reaction forces. With this, they achieve an average speed of 1.3 m/s on flat ground and demonstrated going uphill and downhill, with the ducted fan being instrumental for going uphill and useful for accelerating downhill. However, no slope or velocity metrics were provided. Yang et al. (2024) improves the platform by adding an autonomous navigation system capable of static and dynamic obstacle avoidance, fixed-distance fence following with 7% error, and high-speed skiing up to 7 m/s, all validated on ski slopes. Li et al. (2025) implemented a constrained reinforcement learning controller on the same skiing hexapod, and achieved similar performance but had superior adaptability and manoeuvrability, with a higher range of joint-space utilisation, resulting in tighter turns and overall better handling. However, no metrics were provided for speed, achieved slopes, or energetics.

Zhai et al. (2016) developed a symmetrical hexapod with 3 DoF per leg, each equipped with an internally mounted passive wheel that gets in contact with the ground when the leg is extended. The robot uses cyclic leg-swing trajectories to generate forward propulsion via its passive wheels, achieving a reported skating speed of around 0.4 m/s, which was faster than their baseline tripod gait, but it was noted as less stable. Chitta et al. (2004) also implemented a skate-based propulsion system on a robot with an omnidirectional base (on casters), using two 3-DoF legs equipped with passive wheels to achieve skating motion and directional control. A more recent evolution of Chitta et al. (2004)’s concept was implemented in ALPHRED v2, developed by Hooks et al. (2020), which is a radially symmetrical quadruped robot capable of trotting, hopping, jumping, skating, and bipedal standing for increased height, while also maintaining package pedipulation capabilities. With this multitude of locomotion options, the system achieves good terrain capabilities. For skating, it uses caster wheels for the omnidirectional base and its legs 4 legs for propulsion, achieving speeds of up to 2.1 m/s. The authors state that the limiting factor on speed was the low quality of the wheels rather than the implementation. Hooks et al. (2020)’s system demonstrated the benefits of combining multiple locomotion methods and systems in order to improve the results in real-world deployments.

UC Berkeley developed and open-sourced Jelly^19^ ^,^ ^20^, a quadruped robot that integrates passive wheels mounted on the tibias of its legs. When the robot is in a prone configuration, these passive wheels serve as the sole points of contact with the ground. Additionally, a single actuated wheel is located at the centre of the base and provides propulsion in this mode, effectively transforming the platform into a five-wheeled system. In its fully upright configuration, Jelly operates as a conventional quadruped with standard legged locomotion capabilities. This hybrid design, combining passive and active wheels within a legged system, is particularly interesting, but further details, experimental results, and broader evaluations are needed to thoroughly assess its performance, limitations, and broader applicability.

Hung et al. (2025) developed a tripedal robot featuring an optional central balancing sphere and transformable legs that can switch between casters and full-contact modes. Its locomotion uses offline gait-specific pre-designed trajectories executed via inverse kinematics, with IMU feedback for body orientation and stability. However, no real-time gait adaptation was described. The robot achieved small stair climbing, moving over small obstacles, and stable skating in multiple gaits, with the maximum recorded speed being 0.56 m/s. The robot is also shown skating downhill, but there is no reference to its uphill performance.

Recent research has explored aerial robots equipped with passive wheels, achieving exceptional ground navigation capabilities that significantly extend their operational range while maintaining high speeds (Qin et al., 2020; Zhang et al., 2023; Lin et al., 2024). Li et al. (2026) explored a similar system that is “triphibian”, allowing for aerial, ground and under water navigation. While these systems do not perform true skating locomotion, their hybrid mobility offers noteworthy advantages for versatile navigation scenarios and are worthy of note.

### 2.4 Overview of propulsion methods

The aim of this subsection is to categorise the various propulsion methods presented in the previous sections in more detail.

If a skating robot can steer the direction of its wheels (e.g., a minimum of 4DoF for quadrupeds, or the standard 6DoF biped), there is no need for additional propulsion/control methods, as the dexterous limbs can control the direction and velocity of the robot using motions such as the swizzle gait. However, adding brakes or transformable legs that switch between standard walking and skating modes can provide greater versatility or velocity control to the robot (Bellegarda and Byl, 2019; Endo and Hirose, 2012; Gu et al., 2026).

Underactuated robots (e.g., 3DoF on a quadruped) have a much harder time generating propulsion because the wheels are locked at specific angles, and therefore different propulsion-generating techniques are required. Most of these methods apply to quadrupeds, but some are compatible with other legged configurations (Bjelonic et al., 2018; Cho and Kim, 2024; Chen et al., 2021). The simplest method, from a mechanical perspective, is to use mobility locking (see Figure 2B). By having the wheels mounted at different angles (e.g., on a quadruped, front wheels in-line with the body and the hind legs angled outwards), the mobility of the system is locked without moving or lifting specific legs. This method can be used for high-performance skating as it allows for rapid switching between intense acceleration and energy-efficient cruising. The larger the angle of the wheels (relative to the body x-axis), the better the velocity control, but this comes at the cost of the cruising efficiency. However, at higher wheel angles, there can be considerable tension on the joints, which can lead to failure points or overheating (Cho and Kim, 2024; Chen et al., 2021; Wang et al., 2026).

Claws (see Figure 3) can provide propulsion and braking, but the speeds achieved and velocity control once in motion are poor compared to other approaches. They work by achieving ground contact at specific leg-ground angles, enabling traction but only during particular phases of the gait (Bjelonic et al., 2018; Chen et al., 2023).

Having dedicated push legs is an appropriate solution for multi-legged systems. Performance is highly dependent on the number of legs and stability of the system during push motions (Hung et al., 2025; Yang et al., 2023a). Fan propulsion is also briefly covered in the survey, but more information is needed to thoroughly validate this approach (Yang et al., 2025).

Finally, transformable legs can switch between wheels and standard legs rapidly (see Figures 2A,B). This approach allows for versatility, improved off-road capabilities, and a wide range of possible gaits. However, if all the legs are in the “skating mode”, integrating another propulsion method might be necessary. Additionally, while the configuration has a lot of potential, it can be mechanically complex and can increase the leg mass considerably if not implemented effectively (Hung et al., 2025; Chen et al., 2023; 2024).

## 3 Skating: modelled or learned controllers

Robotic control approaches can be broadly categorized into modelled and learned controllers. Modelled controllers rely on explicit mathematical formulations of the robot’s kinematics and dynamics to compute control actions. They offer strong interpretability and predictable behaviour, but their effectiveness degrades when the underlying models are inaccurate, when operating in highly complex or unstructured environments, or when working with non-linear systems. On the other hand, learned controllers adopt a data-driven approach, most notably reinforcement learning, to directly map state and observations to actions without requiring detailed mathematical models. While this enables greater adaptability to uncertainty and environmental variation, it typically comes at the expense of formal stability and performance guarantees. Moreover, the “black-box” nature of such controllers can hinder interpretability and systematic incremental performance improvement. Hybrid approaches seek to combine the two methods, leveraging the reliability of physics-based modelling while incorporating the flexibility of learning-based methods.

Historically, advanced locomotion for legged robots was mainly achieved using complex modelled controllers, such as Model Predictive Control (MPC) and Zero-Moment Point-based methods (ZMP). However, with the rapid evolution of on-robot computational resources and machine learning techniques, there has been a noticeable shift toward learned controllers, including imitation and reinforcement learning. This transition is largely driven by the ability of learned methods to scale to high-dimensional state spaces, handle complex terrain, generalise to errors and world variability, and exploit large amounts of data to model non-linear, underactuated, or underdetermined robotic systems.

Reinforcement Learning has been extensively explored for both quadrupedal and bipedal locomotion, marking a clear transition away from purely modelled controllers and toward learning-based approaches (Hwangbo et al., 2019; Rudin et al., 2022; 2025; Margolis and Agrawal, 2022; Margolis et al., 2024; Hoeller et al., 2023; Zhuang et al., 2024; Zhang et al., 2025). This shift is not limited to academic research, but is increasingly evident in the commercial sector^21^ ^,^ ^22^. In parallel, advances in simulation fidelity, training pipelines, and reinforcement learning algorithms have significantly accelerated development and experimentation, enabling more scalable and reproducible research workflows (Makoviychuk et al., 2021; NVIDIA et al., 2025; Schwarke et al., 2025; Haarnoja et al., 2018; Schulman et al., 2017; Serrano-Muñoz et al., 2022).

The robotic systems covered in this review similarly reflect an emerging shift in controller design within skating robotics from modelled to learned controllers. As a result, we see higher speeds, improved adaptability to off-road terrain, and greater tolerance for error in dynamically stable gaits. Gu et al. (2026) presented an RL implementation that was deployed on a skating humanoid, demonstrating speeds of up 1.0 m/s, reporting that higher speeds are also possible, while also presenting impressive energy metrics and demonstrating dynamic behaviour in the emergent gait. Han et al. (2026) presented a learned controller for humanoid skateboarding, which demonstrated behaviour more robust than traditional approaches (Takasugi et al., 2016; 2019). Quadrupedal skating robots have also seen many improvements with recent RL implementations. Cho and Kim (2024); Wang et al. (2026) both implemented skating via a learned controller on a Unitree Go1, demonstrating highly dynamic behaviour and increased manoeuvrability, with Wang et al. (2026) going a step further by incorporating energy conservation and training a rapid braking strategy. For the skateboarding quadrupeds, we can observe how the learned controller of Liu et al. (2025) allowed for flat, rough and sloped terrain skateboarding while also maintaining recovery capabilities, demonstrating the adaptability of the method relative to the modelled alternatives. Li et al. (2025) used RL on their skiing hexapod, achieving high speeds and sloped terrain capabilities comparable to their modelled controller (Yang et al., 2023b; 2024) while increasing the platform’s adaptability and manoeuvrability, resulting in better terrain handling and tighter turns.

The adoption of learned controllers in skating robotics remains in its early stages compared to their widespread implementation in conventional legged systems. However, recent advances in both algorithms and computational hardware suggest this trend will continue. While model-based controllers continue to offer value, particularly in their interpretability and predictable behaviour, more high-impact developments may be necessary for them to remain competitive, especially in error-prone and underactuated systems such as the skating robots. The successes demonstrated in recent reinforcement learning implementations across bipedal, quadrupedal, and hexapod skating platforms hint at the potential of learning-based approaches, offering not only superior speed and manoeuvrability but also enhanced adaptability to terrain variability and system uncertainties.

## 4 Discussion

This survey demonstrates that skating locomotion is no longer just a conceptual or experimental platform but a viable and increasingly competitive mode of legged robotic locomotion. Across bipedal, quadrupedal, and other multi-legged platforms, recent implementations consistently show substantial improvements in energy efficiency and achievable speed compared to conventional walking gaits, particularly on smooth or moderately structured terrain. These results confirm that passive wheeled locomotion, when paired with appropriate control strategies, can significantly expand the performance of legged robots.

The surveyed literature also demonstrates that mechanical simplicity does not necessarily result in reduced performance. Passive wheel-legged robots, despite being underactuated and subject to non-holonomic constraints, consistently achieve a lower cost of transport and competitive velocities relative to their standard legged and actively driven counterparts. However, this efficiency comes at the expense of reduced control, requiring the controller to exploit body dynamics, limb coordination, and contact interactions to formulate an effective gait. As a result, configurations with more than 2 legs dominate the skating literature as they provide sufficient redundancy and stability to compensate for underactuation, whereas bipedal skating remains highly sensitive to modelling errors and balance disturbances, with very few examples of truly dynamical bipedal skaters. Even so, bipedal systems are still showing strong recent progress with higher speeds and better generalization compared to previous iterations. Hexapodal systems, particularly those employing skateboard-like skating, demonstrate that additional leg redundancy can further enhance stability, robustness, and achievable speed, but at the cost of increased mechanical and controller complexity.

From a control perspective, the surveyed robots highlight the transition from purely modelled approaches toward learning-based control, particularly reinforcement learning. Model based controllers continue to play an important role in skating locomotion, with ZMP-based planning, centroidal dynamics control, and trajectory optimization still remaining effective at generating stable skating motions under carefully controlled conditions. However, their reliance on accurate models and predefined motion structures limits their scalability to complex terrain, rapid manoeuvres, or changing environmental conditions. In contrast, recent reinforcement learning skating controllers demonstrate a strong capacity to handle underactuated dynamics, friction variability, and contact-rich interactions without explicit modelling of the wheel constraints. Across both humanoid and quadrupedal platforms, learning-based methods consistently achieve high peak speeds, improved adaptability, and more robust performance under uncertainty. These results suggest that RL is particularly well-suited to skating locomotion, where the interaction among limbs, wheels, and the environment is highly nonlinear and difficult to model accurately. Nevertheless, challenges remain in terms of interpretability, formal guarantees, and reproducibility, motivating continued exploration of both model-based and learning-based approaches.

Despite recent advances, several important capabilities remain unexplored. Notably, active braking mechanisms for passive wheels are largely absent from current systems, with few examples in the literature Wang et al. (2007); Naderer et al. (2025); Sun et al. (2026). Most skating robots rely on indirect speed regulation through body posture, gait sequencing, or limb coordination, which limits their ability to perform rapid deceleration and precise speed tracking, and to control safe downhill velocity and traverse off-road terrain. While some platforms employ claws, wheel covers, or transformable feet, these mechanisms typically offer binary or coarse control. The integration of lightweight, adaptive braking represents a promising yet largely unexplored direction that could substantially improve safety and controllability without sacrificing efficiency.

Another challenge across the literature is the lack of unified evaluation metrics. While Cost of Transport (CoT) and maximum velocity are commonly reported, they are often measured under different conditions, terrains, and gait definitions, making direct comparison difficult. Moreover, key aspects such as robustness to disturbances, terrain variability, braking performance, manoeuvrability, and long-term reliability are rarely quantified in a standardized manner. As skating robots move closer to real-world deployment, the absence of consistent benchmarks hinders objective assessment of progress and obscures the true trade-offs between competing designs and control strategies.

Overall, the literature indicates that hybrid skating-legged robots are becoming increasingly feasible, particularly for applications involving long-distance travel in structured or semi-structured terrain. Future research would benefit from a stronger emphasis on integrated system design, combining mechanical innovations such as adaptive braking and transformable limbs with learning-based controllers that exploit these capabilities. Additionally, the development of standardized benchmarks and evaluation protocols would greatly enhance comparability and accelerate progress in this emerging domain.

## 5 Conclusion

This survey reviewed hybrid skating-legged robots with an emphasis on mechanical configurations and the control strategies that enable their locomotion. By examining a broad range of bipedal, quadrupedal, and other multi-legged platforms, the survey shows that skating locomotion combines the adaptability of legged robots with the speed of wheeled locomotion and the energy efficiency of skating. Importantly, this work brings together a previously fragmented body of research spanning different robot morphologies, control methods, and application domains into a single structured survey review.

The papers reviewed demonstrate that passive wheeled legged robots are capable of achieving competitive velocities and considerable reductions in the cost of transport, often with a lower mechanical complexity than actively driven wheel-leg systems. Quadrupedal platforms have emerged as the most practical configuration, offering a balance between stability and underactuation, while bipedal skating remains more challenging due to its sensitivity to balance and modelling errors. Hexapodal skating robots have also demonstrated impressive performance, achieving the highest reported velocities and high robustness due to the increased support polygon size and statically stable gaits. However, their larger mechanical configuration and higher system complexity may limit scalability and adoption compared to quadrupedal designs.

From a control standpoint, there is a clear trend toward learning-based approaches, particularly reinforcement learning, which can also be seen in the broader legged robotics field. While model-based controllers remain effective under well-modelled conditions, learning-based methods consistently exhibit superior adaptability, robustness to uncertainty, and performance. Recent results suggest that reinforcement learning is especially well suited to the complex limb-wheel environment interactions specific to skating locomotion.

Despite this progress, key challenges remain. Adaptive braking mechanisms for passive wheels are largely absent, limiting precise speed control and safe high-speed operation. Additionally, the lack of unified evaluation metrics makes objective comparison across platforms difficult. Addressing these gaps through integrated mechanical design, learning-based control, and standardised benchmarks would be critical for advancing skating legged robots toward reliable real-world deployment.

## Footnotes

1 ETH’s ANYmal on Wheels: https://rsl.ethz.ch/robots-media/anymal-wheels.html.

2 Boston Dynamics’ Handle: https://www.youtube.com/watch?v=-7xvqQeoA8c.

3 PRISMA: https://www.prisma-statement.org/.

4 Inciteful: https://inciteful.xyz/.

5 YouTube video of Sony QRIO skating: https://www.youtube.com/watch?v=-_GxmkWc7WU.

6 IEEE Spectrum article on 2018 Ski Robot Challenge: https://spectrum.ieee.org/ski-robot-challenge.

7 Another article on the 2018 Ski Robot Challenge covering more details: https://www.koreaherald.com/article/1586648.

8 Unitree G1: https://www.unitree.com/g1.

9 Video #1 of the RollerWalker skating: https://youtu.be/P5ay5VC9ba4?si=HHF-14_vXi-W_uZW.

10 Video #2 of the RollerWalker skating: https://youtu.be/R86a_8M5SBw?si=lg2BKc_x-8oZUgEn.

11 Snake Monster - RollerWalker like skating: https://youtu.be/Vhgft53qfDQ?si=dQJtfUdMww_FJXlx.

12 ANYmal skating: https://youtu.be/fJfAWiylpxw?si=SmNji9t89U8ZuM40.

13 Skaterbots skating: https://www.bbc.co.uk/future/article/20190208-the-robot-that-learned-how-to-skate-on-ice.

14 Demonstration of skating gait on Chen et al. (2021), Chen et al. (2022) prototype: https://youtu.be/BbWUmn5_IYw?si=BTC3PiweMqMPTWdN.

15 QSkater-E skating: https://youtu.be/ShYGhkTHYOg?si=pinkUd8nz2NV553Q.

16 Unitree Go1: https://tinyurl.com/unitreego1.

17
Cho and Kim (2024)’s Go1 skating: https://youtu.be/bjbmjXcArHo?si=auM4Ewb7cCk1aNAL.

18 Boston Dynamics’ Spot skating: https://youtu.be/LMPxtcEgtds?si=1aooAlrOPeQinIan&t=22.

19 Jelly presentation video: https://www.youtube.com/watch?v=9sITXoZu2gY.

20 Webpage discussing Jelly and repository links: https://amaberkeley.github.io/webpage/.

21 Boston Dynamics’ Atlas shift to RL: https://bostondynamics.com/blog/flipping-the-script-with-atlas/.

22 Spot and RL: https://bostondynamics.com/blog/starting-on-the-right-foot-with-reinforcement-learning/.
